# Changes in the Fecal Microbiota Associated with a Broad-Spectrum Antimicrobial Administration in Hospitalized Neonatal Foals with Probiotics Supplementation

**DOI:** 10.3390/ani11082283

**Published:** 2021-08-02

**Authors:** Francesca Freccero, Aliai Lanci, Jole Mariella, Elisa Viciani, Sara Quercia, Andrea Castagnetti, Carolina Castagnetti

**Affiliations:** 1 Department of Veterinary Medical Sciences, University of Bologna, Via Tolara di Sora 50, Ozzano Dell’Emilia, 40064 Bologna, Italy; francesca.freccero2@unibo.it (F.F.); jole.mariella2@unibo.it (J.M.); carolina.castagnetti@unibo.it (C.C.); 2 Wellmicro srl, Via Piero Gobetti 101, 40127 Bologna, Italy; elisa.viciani@wellmicro.com (E.V.); sara.quercia@wellmicro.com (S.Q.); andrea.castagnetti@wellmicro.com (A.C.); 3 Health Science and Technologies Interdepartmental Center for Industrial Research (HST-ICIR), University of Bologna, Via Tolara di Sopra 41/E, Ozzano dell’Emilia, 40064 Bologna, Italy

**Keywords:** fecal microbiota, antimicrobials, dysbiosis, foals

## Abstract

**Simple Summary:**

Post-antibiotic intestinal dysbiosis leads to an overall reduction in bacterial and functional diversity, along with a minor resistance against pathogens. The study aimed to determine the changes on the fecal microbiota in hospitalized neonatal foals administered with broad-spectrum antimicrobials and supplemented probiotics. Fecal samples were collected at hospital admission, at the end of the antimicrobial treatment and at discharge. Seven foals treated with intravenous ampicillin and aminoglycosides for a mean of seven days were included. The results suggest that the fecal microbiota of neonatal foals rapidly returns to a high diversity after treatment. While the findings need to be confirmed in a larger population, the study suggests that in foals, the effect of antimicrobials may be strongly influenced by the changes that occur over time in the developing gut microbiota. Of note, the findings are influenced by the use of probiotics, and whether the changes would be consistent in antimicrobial-administered but not supplemented foals remains to be elucidated.

**Abstract:**

There is a wide array of evidence across species that exposure to antibiotics is associated with dysbiosis, and due to their widespread use, this also raises concerns also in medicine. The study aimed to determine the changes on the fecal microbiota in hospitalized neonatal foals administered with broad-spectrum antimicrobials and supplemented probiotics. Fecal samples were collected at hospital admission (Ta), at the end of the antimicrobial treatment (Te) and at discharge (Td). Feces were analysed by next-generation sequencing of the 16S rRNA gene on Illumina MiSeq. Seven foals treated with IV ampicillin and amikacin/gentamicin were included. The mean age at Ta was 19 h, the mean treatment length was 7 days and the mean time between Te and Td was 4.3 days. Seven phyla were identified: Actinobacteria, Bacteroidetes, Firmicutes, Fusobacteria, Proteobacteria, TM7 and Verrucomicrobia. At Ta, Firmicutes (48.19%) and Proteobacteria (31.56%) were dominant. The alpha diversity decreased from Ta to Te, but it was the highest at Td. The beta diversity was higher at Ta than at Te and higher at Td than at Te. An increase in *Akkermansia* over time was detected. The results suggest that the intestinal microbiota of neonatal foals rapidly returns to a high diversity after treatment. It is possible that in foals, the effect of antimicrobials is strongly influenced or overshadowed by the time-dependent changes in the developing gut microbiota.

## 1. Introduction

Neonatal foals are predisposed to life-threatening bacterial infections, and antimicrobials are routinely and widely employed in equine neonatal medicine, either prophylactically or therapeutically [1]. Sepsis is the most common cause of death during the first week of life and failure of passive transfer of immunity (FPT) is a leading risk factor [1]. Ischemic-hypoxic intestinal damage also puts foals affected from perinatal asphyxia syndrome (PAS) at higher risk of developing sepsis [2]. In the treatment or prevention of sepsis, the empirical selection of broad-spectrum antimicrobials, preferentially administered by intravenous (IV) route, is paramount in most cases. A combination of beta lactams and an aminoglycoside is the most commonly used as ampicillin 22–30 mg/kg IV or orally (PO) every 6–8 h and amikacin 25 mg/kg IV every 24 h [3]. This combination has been recently shown to be effective in 91.5% of bacterial isolates in neonatal foals [2,4].

While the use of perinatal broad-spectrum antibiotics is common, there is wide evidence that early exposure to antibiotics is associated with dysbiosis and long-term impacts on microbial diversity and health, with derangement in the development of the immune, metabolic and nervous system in humans [5,6,7]. Dysbiosis means an imbalance, qualitative and/or quantitative, in the intestinal microbiota composition [8]. Antimicrobials not only affect the target pathogens, but they bring collateral effects over the entire microbial ecosystem [9]. Pharmacological agents damage bacterial cells, causing loss of membrane polarity and integrity and a reduction of nucleic acid content; genic expression, protein synthesis and metabolome are also affected [9,10].

Antimicrobial-associated dysbiosis leads to an overall reduction in bacterial and functional diversity, along with a minor resistance against pathogens [9,11] and the establishment of favorable conditions for opportunistic bacteria to grow [12]. The potential antimicrobial induction and selection for resistant, or able to acquire resistance, microorganisms are an increasingly relevant issue [13]. Indeed, microbiota respond to the insult early, increasing the expression of a small number of resistances encoding genes [9].

The diverse effect on bacterial taxa is strikingly dependent on the antimicrobials classes and combinations used. This effect may be due not only to the nature of the agent itself, but also depends on other factors including dose and route, period of exposure, pharmacokinetics, pharmacodynamic and the stability of the microbiota itself [6,9,14,15,16]. In this light, while antimicrobials susceptibility testing is commonly employed in the practice and remains a mainstay for appropriate drug selection. The pharmacodynamics of the drugs should be further considered in the choice of the individual antimicrobial administration [17].

Nevertheless, the gut microbiota has a variable degree of resilience, which allows recovering to pre-treatment conditions after the balance has been disrupted. However, studies suggest that this recovery might not be complete at least over a long period [6], and this has been shown in horses as well [18,19].

Antimicrobial-associated dysbiosis also raises concerns in equine neonatal medicine. In foals, the development of post-natal microbiota has been investigated by Next Generation Sequencing (NGS), and it is characterized by a high instability and rapid changing over time [20,21,22,23,24]. Costa et al. [25] suggested that such dynamicity might prompt a more rapid recovery respect to adult horses, even after external perturbations, such as antimicrobials.

With respect to culture-dependent techniques, molecular approaches dramatically improved the ability to examine gut microbiota composition and dynamics related to antimicrobials administration [7].

A few reports exist exploring the effects on gut microbiota of a broad-spectrum antimicrobial therapy in adult horses [20,26,27,28]. Antimicrobial resistance is also an alarming and emerging challenge in equine medicine [17]. In this contest, in foals, antimicrobial resistance gene profiles [29] and emergence of multi-drug resistant Rhodococcus equi due to wide-spread use of a macrolide–rifampin combination [30] has been investigated by NGS. To the authors’ knowledge, no studies to date have reported changes in gut bacterial microbiota in relation to broad-spectrum antimicrobial use in neonatal foals. Alterations of gut microbiota after antimicrobial administration have been reported in adult horses [19], and early exposure to antimicrobials has been associated with long term implications in human babies [2]. This information prompts the need for deep insights on the antimicrobials’ effects on foals’ intestinal microbiota in view of a more informed drug selection.

This study aimed to determine changes related to broad-spectrum antimicrobials administration on the fecal microbiota (FM) of hospitalized neonatal foals by means of NGS of the 16S rRNA gene. Fecal microbial ecosystem was characterized with regard to relative abundance at mean and individual levels before and after administration of a broad-spectrum antimicrobial treatment in foals hospitalized for common neonatal pathologic conditions (as sepsis, prematurity/dysmaturity and Perinatal Asphyxia Syndrome). Moreover, biodiversity changes in gut microbiomes and their relationship with the antimicrobial treatment were analyzed.

## 2. Materials and Methods

### 2.1. Animals and Samples Collection

The study was conducted at the Equine Perinatology Unit (EPU) of the Veterinary Teaching Hospital of the University of Bologna during the foaling season 2019.

All procedures on the animals were carried out according to the ethics review committee at the University of Bologna. Oral informed consent was given by the owner for each animal.

Fecal samples were collected from hospitalized neonatal foals administered a broad-spectrum antimicrobial treatment either therapeutically or prophylactically. At enrolment, foals should have no history of antimicrobials administration, and dams of the foals should have not received antimicrobials in the last three months. Enrolled foals were either born from mares hospitalized at the EPU for attended delivery or hospitalized after birth.

Pregnant mares were hospitalized at about 310 days of gestation. They were judged clinically normal based on daily physical examination, complete blood count (CBC) and serum biochemistry at admission, and periodical ultrasonographic monitoring of feto-placental wellbeing was conducted upon foaling. Foals received a full diagnostic protocol including: full physical examination, CBC and biochemistry, arterial blood gases analysis, serum IgG and blood lactate concentration and blood culture. Foals born at the EPU were also assessed at birth by means of an Apgar score [31].

Diagnostic criteria were defined as follows.

Failure of passive transfer of immunity was diagnosed as blood IgG concentration < 800 mg/dL after 18 h of life [32].

Foals were classified as septic in the presence of both infection and systemic inflammatory response [33]. Infection was confirmed on the basis of one of the following:

- positive blood culture;
- positive culture of samples from local sites of suspected infection;
- postmortem examination. Systemic inflammatory response syndrome (SIRS) diagnosis was made as suggested by Wong and Wilkins [34].

Foals were classified as affected by Perinatal Asphyxia Syndrome (PAS) on the basis of clinical history (i.e., abnormal pregnancy and/or parturition), clinical signs (i.e., non-infectious and non-congenital neurological conditions) and laboratory abnormalities [35].

Foals were defined as premature if born prior to 320 days gestation and were defined as dysmature if born after 320 days but with immature physical characteristics (e.g., low body weight and inability to maintain body homeostasis) [33,36]. Foals were individually treated and monitored according to their clinical condition. All the foals received antimicrobials intravenously through a catheter in the jugular vein and received a probiotic supplementation orally. Other treatments included prophylactic gastric therapy and fecal microbial transplantation (FMT) where indicated on individual basis.

According to clinical management, foals and their dams were housed in single, wide, straw-bedded boxes, either together or separated by a gate allowing contact, and foals were fed mare’s milk and/or milk replacer during the hospitalization. They were turned out during the day only where applicable. Mares were fed hay ad libitum and 14–16% sweet feed concentrates.

Fecal sampling was planned at three time points:

- before the start of antimicrobial treatment (Ta, admission);
- at the end of the treatment (Te);
- the day of discharge from the hospital (Td).

At least 2 g of feces were collected from the rectal *ampulla* and from the center of the fecal material [23]. The samples were immediately stored in sterile vials at −25 °C and subsequently transferred at −80° C.

To the study purposes, the following data were recorded: diagnosis, age (hours) at the start of antimicrobial treatment, antimicrobial treatment duration (days), time from the end of treatment to the hospital discharge (Te–Td; days), antimicrobials (dosage and route), occurrence of diarrhoea and further treatments (including probiotics, prophylactic gastric therapy, FMT). Moreover, source of milk feeding (mare’s milk, milk replacer, both) during the observation period was recorded for each foal.

### 2.2. Bacterial DNA Extraction and 16S rRNA Sequencing

Bacterial DNA was extracted from stools with a DNeasy Blood & Tissue kit (QUIAGEN, Hilden, Germany) as previously described by Biagi et al. [37], adding three 1-min bead-beating steps with four 3-mm glass beads and 0.5 g of 0.1-mm zirconia beads (BioSpec Products, Bartlesville, OK, USA) in a FastPrep instrument (MP Biomedicals, Irvine, CA, USA) at 5.5 movements per second [38]. Two microliters of DNase-free RNase were added, and samples were incubated at 37 °C for 15 min. The supernatant was treated with proteinase K and DNA purified using the QIAamp Mini Spin columns (QUIAGEN, Hilden, Germany) following the manufacturer’s instructions.

DNA samples were quantified using the NanoDrop ND-1000 (NanoDrop Technologies, Wilmington, DE, USA).

The V3–V4 region of the 16S rRNA gene was amplified by means of the primer set S-D-Bact-0341-b-S-17/S-D-Bact-0785-a-A-21 [39] carrying Illumina (Illumina, San Diego, CA, USA) overhang adapter sequences, as previously described [40].

Amplicons were purified with a magnetic bead-based clean-up system (Agenetcourt AMPure XP, Beckman Coulter, Brea, CA, USA). Indexed libraries were prepared by limited-cycle PCR using Nextera technology and further cleaned up again by the magnetic bead-based clean-up system. Final libraries were pooled at 4 nM, denatured and diluted to 6 pM before loading onto the MiSeq flow cell (Illumina, San Diego, CA, USA). Sequencing was performed by a 2 × 300 bp paired end protocol, according to the manufacturer’s instructions (Illumina, San Diego, CA, USA) [41].

Amplicon sequences were deposited in the MG-Rast database (http://metagenomics.anl.gov/mgmain.html?mgpage=project&prject=59129e34be6d67703833303632 accessed on 12 September 2019).

### 2.3. Bioinformatics and Statistics

Raw sequences were analyzed using Quantitative Insights Into Microbial Ecology (QIIME) version 1.9.1 [42]. Reads were filtered for quality using the split_library_fastq.py script of the QIIME pipeline with default values. High-quality reads were clustered into Operational Taxonomic Units (OTUs) at 97% homology [43]. All singleton OTUs were discarded to avoid chimeric sequences. The filtering of chimeric OTUs was performed by using ChimeraSlayer [44]. OTUs were taxonomically assigned using RDP classifier (Ribosomal Database Project (RDP) database (http://rdp.cme.msu.edu/ accessed on 12 September 2019) against Greengenes database from May 2013 release (http://greengenes.secondgenome.com/downloads) (“Greengenes:16S rDNA data and Tools”, 2011).

For the OTU sharing analysis, OTUs representing more than 0.01% of the total OTU number were considered.

The bacterial abundance data were imported into R (version 3.6.1, R Foundation for Statistical Computing, Vienna, Austria. URL http://www.R-project.org/) on Rstudio v1.1.456 (RStudio Team (2020). RStudio: Integrated Development for R. RStudio, PBC, Boston, MA URL http://www.rstudio.com/) where all statistical analyses were performed. A *p*-value < 0.05 after False Discovery Rate (FDR) correction was considered as statistically significant. Alpha diversity was evaluated by calculating Observed species, Chao1, Shannon, ACE (Abundance-based Coverage Estimator), Inverse Simpson’s and Fisher’s indices using R package phyloseq [45,46]. The difference of alpha diversity was evaluated using ANOVA and Tukey’s HSD (honestly significant difference) tests for normally distributed data or Wilcoxon–Mann–Whitney with Holm–Bonferroni correction method for non-normally distributed data. To compare microbial composition between samples, beta diversity was measured by calculating the Bray-Curtis distance matrix of weighted and unweighted UniFrac. Principal coordinates analysis (PCoA) was applied on the distance matrices to generate bi-dimensional plots in R. Dispersion of the PCoA clusters was compared using the betadisper function in R vegan package [47]. The permutation analysis of variance (PERMANOVA) test, calculated using the function adonis in the vegan package (CRAN Package vegan; [48]), was performed to determine whether there was a significant separation between different sample groups. The plots were graphed using ggplot2 R packages [49]. To identify taxa that were significantly different between sample groups, environmental variable superimposition was performed on the study subject multidimensional scaling (MDS) plot using function envfit contained in package vegan of R studio. In order to test for the differential abundance of taxa that could drive the differences observed between inferred microbial communities derived from the different samples, we performed DESeq2 analyses [50].

## 3. Results

Seven hospitalized neonatal foals that had received the same association of antimicrobials (ampicillin combined with an aminoglycoside IV) and had a complete sampling series were available for analysis.

The demographic and clinical data regarding the individual foals are presented in Table 1. Three out of the seven foals were hospitalized after birth, while four out of seven were born at the EPU, all between March and June. Two were males and five females.

The mean age at Ta was 19 h (0–42 h). All seven foals received ampicillin (50 mg/kg IV QID) combined with an aminoglycoside (amikacin 25 mg/kg IV SID in five out of seven foals, or gentamicin 12 mg/kg q 36 h in two out of seven foals), therapeutically or prophylactically, according to their diagnosis. One foal (CM) had a diagnosis of sepsis (*E. coli* isolated from blood culture), dysmaturity and FPT. Two foals were treated prophylactically for PAS, three foals for FPT and one for PAS and FPT.

The mean length of the antimicrobial treatment was 7 days (5–12 d), and the mean time between Te and Td was 4.3 days (1–9 d).

The treatments and management were tailored on an individual basis according to the clinicians’ judgement. All the foals received probiotics (Yovis, Alphasigma spa, Bologna, Italy; 1–3 g/day) for the duration of the antimicrobial treatment. As shown in Table 1, five of the seven foals received prophylactically omeprazole (2 mg/kg PO SID) and/or sucralfate (15 mg/kg PO TID) during the hospitalization period.

Four foals developed a mild diarrhea, which was not further investigated, without major complications: three during the antimicrobial treatment (CM, GRA, TR) and one after the end of the treatment (FDT). Three foals received a fecal microbial transplantation (FMT) during the diarrhea; in two of the three cases, the donor was the dam, and in one case, the donor was a foster mare. The diarrhea did resolve in all cases within hospital discharge.

Two of the seven foals received a milk replacer along with dam’s milk during the observation period; one orphan foal (TR) received milk replacer and milk from a foster mare, and feces from the same foster mare were provided for coprophagy. All the other foals had access to their dams’ feces during the hospitalization.

All foals were diagnosed as clinically normal at discharge from the hospital.

### 3.1. Analysis of the Composition of Gut Bacterial Microbiota in Relation to Antimicrobial Treatment

The analyzed samples included 4 samples of meconium at Ta (CM, GRA, FDT, GR) and 17 fecal samples at Ta, Te and Td. Sequencing of the 16S rRNA gene yielded 188.027 high-quality reads (mean ± SD per sample 5,530,206 ± 1,929,203).

All bacterial OTUs detected at a relative abundance greater than 2% were figured in bar plots of fecal microbiota composition of the foals at the three timepoints in relation to the antimicrobial therapy. The remaining OTUs were classified as “other” and unclassified bacteria group under the definition “unassigned_other”. All bacterial OTUs with their relative abundance detected at the three timepoints are reported in Table S1.

Mostly, no significant differences were found in the relative abundances between timepoints at the family nor at the genus level, and the results are reported as trends.

**Figure 1:**
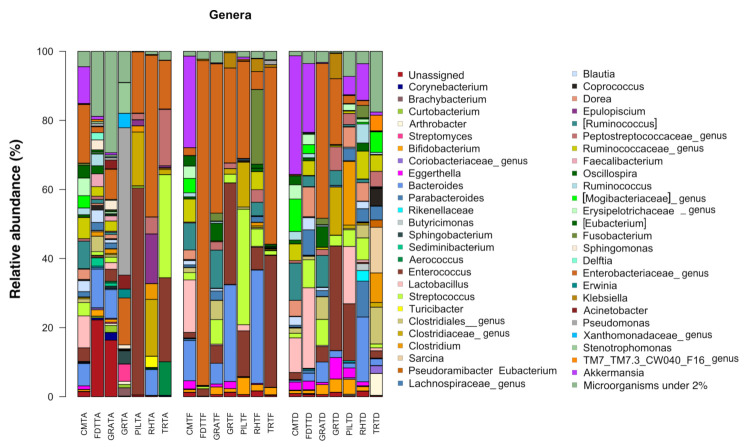
Compositional structure of the gut microbial communities in seven hospitalized neonatal foals before and after broad-spectrum antimicrobial treatment. Bar plots represent the relative abundance at genus level in fecal samples at the timepoints Ta, Te and Td. Genera under the threshold of 2% of relative abundance were clustered as “other”, and unclassified genera were clustered as “Unassiged”. No significant differences were found between timepoints (*p* ≥ 0.05). Samples are identified by acronyms of foals’ names followed by sampling time. A: before antimicrobial treatment; F: end of antimicrobial treatment, D: time of hospital discharge.

The relative abundance of bacterial OTUs at genus levels in the seven foals across three timepoints are plotted in Figure 1.

#### 3.1.1. Characterization of Bacterial Ecosystem before Antimicrobial Treatment (Ta)

Bacterial OTUs were clustered into seven different phyla: Firmicutes (48.1%), Proteobacteria (31.56%), Bacteroides (7.55%) e Actinobacteria (4.48%), Verrucomicrobia (1.79%), with TM7 and Fusobacteria (less than 1%) (Table S1).

At a family level, Enterobacteriaceae and Enterococcaceae had the more represented mean relative abundance (17.55% and 13.23%), followed by a decreasing contribution of Lachnospiraceae (7.07%), Pseudomonadaceae (6.40%), Clostridiaceae (6.29%), Streptococcaceae (5.32%), Bacteroidaceae (4.78%) and Ruminococcaceae (3.80%). At a genera level, genera owing to Enterobacteriaceae (17.08%) and *Enterococcus* (13.23%) were the most prevalent. Other taxa included were *Pseudomonas* (6.33%), *Streptococcus* (5.30%), *Bacteroides* (4.76%), genera within the family Clostridiaceae (4.90%) including *Clostridium*, Peptostreptococcaceae (3.56%) and *Epulopiscium* (2.40%). Genera of the *Akkermansia* and *Lactobacillus* and Ruminococcaceae families were identified at a relative abundance < 2%.

#### 3.1.2. Characterization of Bacterial Ecosystem at the End of Antimicrobial Treatment (Te)

In comparison to Ta, fecal samples collected at Te showed a trend of reduction in the phyla Firmicutes (38.93%) and Actinobacteria (3.03%), which were replaced by an increment of Verrucomicrobia (3.95%), Fusobacteria (3.44%), Bacteroidetes (11.37%) and Proteobacteria 11.44%) (Table S1).

On a family level, a loss of microbial diversity was noted. In particular, the main changes were an increase in Enterobacteriaceae (37.64%; *p* = 0.02) and a tendency to increase in Bacteroidaceae (11.21%; *p* = 0.29) and Verrucomicrobiaceae (3.94%; *p* = 0.37), along with a trend to reduce in Clostridiaceae (2.45%; *p* = 0.20), Pseudomonadaceae (0.19% *p* = 0.33)—which almost disappeared—and Mogibacteriaceae (*p* = 0.34).

Coming to genera, a trend to reduction in the overall microbial diversity was still evident, along with a prevalence in the relative average abundance of members of the Enterobacteriaceae (35.94%). Genera such as *Bifidobacterium* (1.83%; *p* = 0.15), *Bacteroides* (11.21%; *p* = 0.27), *Fusobacterium* (3.44%; *p* = 0.33), *Akkermansia* (3.94%; *p* = 0.32) and *Streptococcus* (7.44%; *p* = 0.55) were all represented with a tendency to increase in average abundance.

#### 3.1.3. Characterization of Bacterial Ecosystem at the Time of Hospital Discharge (Td)

Samples at Td showed a trend to an increased mean relative abundance in the phyla Firmicutes (61.58%) at most, Verrucomicrobia (10.28%) and Actinobacteria (6.35%), while it was lower for Proteobacteria (11.44%), Bacteroidetes (7.66%) and Fusobacteria (0.82%) compared to Te. (Table S1).

The families Lachnospiraceae (13%; *p* = 0.035), Verrucomicrobiaceae (10.22%; *p* = 0.06), Clostridiaceae (8.32%; *p* = 0.13) and Mogibacteriaceae (2.69%; *p* = 0.3) tended toward a higher mean abundance with respect to Te; on the contrary, Enterobacteriaceae (10.85%; *p* = 0.09) and Bacteroidaceae (4.72%; *p* = 0.16) were less represented.

At a genera level, the mean relative abundance of *Akkermansia* (10.22%; *p* = 0.069)*, Lactobacillus* (7.41%)*, Ruminococcus* (4.16%; *p* = 0.18)*, Clostridium* (3.90%) and *Bifidobacterium* (1.99%; *p* = 0.15) showed a tendency to increase compared to Te, while the genera *Bacteroides* (4.67%; *p* = 0.15), *Enterococcus* (7.98%; *p* = 0.30) and *Streptococcus* (4.61%; *p* = 0.55) tended to be lower than Te.

Finally, when microbiota composition was analyzed throughout time, DeSeq analysis detected a significant increase in *Akkermansia* (*p*-value FDR-corrected = 9.78 × 10^−31^) and members of the Mogibacteriaceae (*p*-value FDR-corrected = 3.03 × 10^−10^) after antibiotic treatment, while the family Enterobacteriaceae showed later, between the end of therapy and the time of discharge, only a trend to increase.

### 3.2. Alpha Diversity Analysis of the Bacterial Ecosystem in Relation to Antimicrobial Treatment

**Figure 2:**
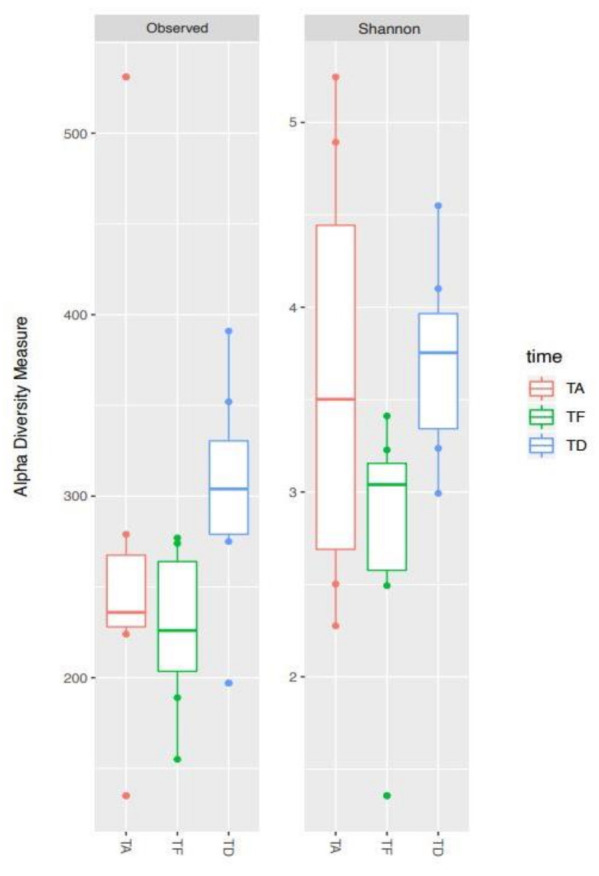
Alpha diversity changes of gut microbial ecosystem in seven hospitalized neonatal foals before and after broad-spectrum antimicrobial treatment. Boxplots of observed species and Shannon indices representing data at the timepoints Ta (TA), Te (TF) and Td (TD).

In order to compare the alpha diversity of the microbial ecosystems among foals at the three timepoints, the richness indices Observed species and Shannon were plotted (Figure 2).

Species richness was variable across samples at all the three timepoints. Overall, Te showed a trend toward a lower level of biodiversity compared to Ta. Afterwards, albeit not significantly, Td appeared to be the most diverse, with a species richness tending to be higher than the initial condition (see, in particular, the Observed species index, *p*-value FDR-corrected = 0.052).

The box represents the interquartile ranges (IQR) containing 50% of the samples, the horizontal line in a box represents the median, whiskers represent maximum and minimum within 1.5 × IQR and circles represent outliers. No significant differences were found between timepoints (all *p* ≥ 0.05).

Ta: before antimicrobial treatment, Te: end of antimicrobial treatment, Td: time of hospital discharge.

### 3.3. Beta Diversity Analysis of the Bacterial Ecosystem in Relation to Antimicrobial Treatment

**Figure 3:**
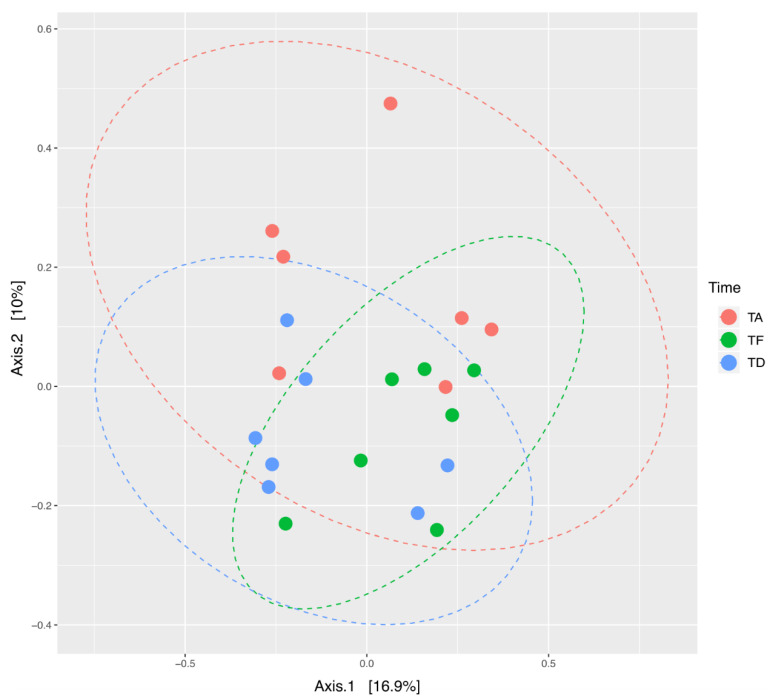
Beta diversity changes of gut microbial ecosystem in seven hospitalized neonatal foals before and after broad-spectrum antimicrobial treatment. PCoA analysis on unweighted UniFrac distances of fecal microbiota at the three timepoints. Different colors represent samples at three timepoints. A significant difference in clustering of samples at the three timepoints was found (PERMANOVA = 0.05). TA (Ta): before antimicrobial treatment, TF (Te): end of antimicrobial treatment, TD (Td): time of hospital discharge.

The Principal Coordinates Analysis showed the highest inter-individual beta diversity at Ta. It resulted considerably reduced at Te, so that samples clearly clustered together with a more limited dispersion in the PCoA graph (Figure 3). This result suggests that, as a consequence of antimicrobial therapy, microbial communities tend to resemble more to one another across foals. Subsequently, already a few days after the end of the treatment (Td), the distance between samples was increased, meaning that the individual bacterial ecosystems started to diverge again as the foals were rebuilding their gut microbiota. Nevertheless, a greater convergence at Td than Ta was depicted.

**Figure 4:**
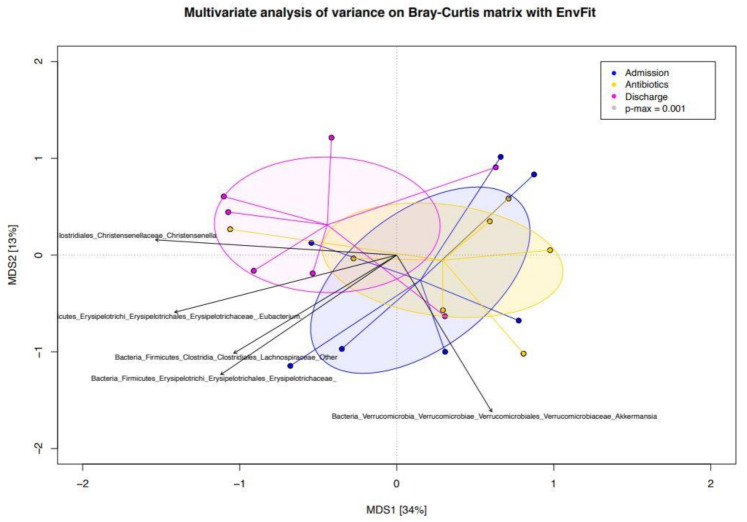
PCoA plot based on the Bray–Curtis dissimilarity index of the genus-level compositional structures of the gut microbiota at the three timepoints in hospitalized neonatal foals. Bacterial microorganisms are superimposed and the bacterial genera most contributing to the ordination space are depicted. Direction of the vectors indicates increasing gradient, and length is proportional to the correlation strength. Admission (Ta): before antimicrobial treatment, Antibiotics (Te): end of antimicrobial treatment, Discharge (Td): time of hospital discharge. MDS: multidimensional scale.

In Figure 4, the bacterial genera were superimposed on the PCoA plot based on the Bray–Curtis dissimilarity index of the compositional structures of samples at the three timepoints, and the bacterial genera most contributing to the ordination space were depicted. Fibrolytic microorganisms, such as *Eubacterium*, were more characteristic of Ta, but then it was lost after the treatment to reappear at discharge. The genus *Akkermansia* was more characteristic of Td than the earlier time points.

## 4. Discussion

In this study, we aimed to evaluate by means of NGS the impact of a broad-spectrum antimicrobial treatment by intravenous route on the gut bacterial microbiota in hospitalized newborn foals. To date, there are no similar studies in neonatal foals for direct comparison. The findings will be discussed in the context of recent literature on antimicrobials-associated gut microbiota changes in adult horses and other species explored by means of NGS technology.

### 4.1. Fecal Microbiota in Neonatal Foals after IV Broad-Spectrum Antimicrobials

Empirical first line broad-spectrum treatment at our hospital regularly includes sodium-ampicillin—provided market unavailability of IV salts of penicillin G—mostly combined with amikacin, which is usually preferred to gentamicin for its lower reported nephrotoxicity and greater efficacy [2]. Albeit amikacin and gentamicin are not completely overlapping in their spectrum of action [1], they were considered together as a class to the aim of the present study. While dosage and route were standardized during the study, it was not possible to do so with the treatment duration. The duration of treatment was not homogenous, potentially causing a different pressure and impact on microbial taxa in the different subjects. Nevertheless, in the Authors’ opinion, a general trend of changes toward a loss of microbial diversity after antimicrobials administration would be expected similar to other models.

Indeed, data from the present study show that the use of this antimicrobial association in hospitalized neonatal foals reduces alpha diversity and causes a strong clusterization of beta diversity of the gut bacterial communities after the treatment (Te). Indeed, a qualitative reduction of microbial communities was observed across the samples. The results from PCoA analysis show that at Te, the foals’ gut microbial ecosystems inter-individual variability is smaller when compared to Ta or at discharge (Td). The picture is in agreement with the literature, showing that any antimicrobial will have an effect on the overall host flora—the intestinal flora in particular—and not only on the bacterial target [51].

The perturbation of gut microbial communities depends on antimicrobial class, dose and period of exposure, as well as their pharmacological action on the target bacteria [16]. In adult horses, Costa et al. [20] have shown how different antimicrobial treatments have an impact on fecal microbiota. In particular, the effect on bacterial communities’ richness and diversity was the highest with oral trimethoprim-sulfadiazine (TMS) administration respect to IV penicillin or ceftiofur. A route-dependent higher intestinal concentration and a broader spectrum of action of TMS have been hypothesized as the reason, albeit this remains to be investigated. Nevertheless, it has been demonstrated that IV-administered antibiotics are able to achieve high intestinal concentration in other species [20]. Only one broad-spectrum IV antimicrobial combination was investigated in the present study, thus the effect on fecal bacterial microbiota of different routes or combinations is unknown.

Interestingly, the most profound impact on fecal microbiota has been reported from days 2 to 5 in adult horses when different antimicrobials are administered [20,27,28]. Albeit we did not perform a sequential sampling, this is comparable with the earliest time at which an effect was detected in our study (two foals that received 5 days of treatment). Similarly, in human babies, a dramatic impact on fecal microbiota has been detected already within a few days of antibiotic administration [7]. In particular, in preterm infants, a combination of ampicillin and gentamicin administered for 5–7 days in the first three weeks of life produced a strong reduction in microbial diversity, while the effect was less sustained in case of a shorter course (1–4 days) [52].

When the FM composition in terms of the taxa’s relative abundance was observed, a large inter-individual variability characterized the microbial ecosystems during the whole study period. Some trends of change were identified comparing samples before and after antimicrobial treatment. A tendency to a decrease for Firmicutes and increase for Verrucomicrobia after the treatment is in line with reports in humans, where these phyla represent, overall, the most sensitive and resistant phyla to antimicrobials, respectively [9]. Moreover, similar changes in Firmicutes and Verrucomicrobia have been reported after beta lactams administration in a murine model [8]. Partially similar to our findings, the combination of ampicillin and gentamicin causes alterations in the phyla Actinobacteria, Bacteroidetes, Firmicutes and Proteobacteria in humans [9].

A decrease in Verrucomicrobia was similarly found after 3 days of oral metronidazole in adult horses [27]. In contrast to our findings, Costa et al. [20] found an opposite direction of changes in Firmicutes and Verrucomicrobia after 5 days of oral TMS, but not parenteral penicillin or ceftiofur, suggesting that any antimicrobial has a specific effect on gut microbiota communities. Indeed, it is acknowledged in human literature that different antibiotic treatments have diverse effects on the general composition of IM, as well as on particular microbial taxa [9]. This effect may be due not only to the antibiotic itself or the cocktails used, but to other factors including dose and route, period of exposure, pharmacokinetics and resistance or transformation of the agent by each microbe [9,16].

In the present study, the phylum Verrucomicrobia was dominated by the genus *Akkermansia*, which showed a significant increase in abundance, in line with its mentioned resilience. Indeed, *Akkermansia* has been shown to appear early in the gut microbiota and increase throughout time in newborn foals [20,23,24], as well as in infants mainly through breast-feeding [53,54]. *Akkermansia* is associated with intestinal integrity, and its metabolic activity influences short-chain fatty acids (SCFA) production contributing to the gut health [55].

In this study, the only significant change was the higher abundance of the members of Enterobacteriaceae after antimicrobial treatment. This change is consistent in the human literature, where an increase in Enterobacteriaceae—*Enterobacter* spp., in particular—has been reported in babies receiving early antimicrobials, and more specifically, the combination of ampicillin and gentamicin [6,11,16,52,56]. Furthermore, the same combination proved a similar effect on gut microbiota in murine models [8].

A reducing trend in *Faecalibacterium* and *Blautia* was found, similarly to findings induced by the same antibiotics’ association in murine models [8]. These genera are well known for their beneficial role in gut health and function through short-chain fatty acid production and amelioration of inflammation and figure among the most sensitive to a number of different antimicrobials and combinations in humans [9].

In opposition with the human literature, *Bifidobacterium* and *Bacteroides* tended to be more abundant after treatment. These two genera have been reported as being sensitive to the highest number of antimicrobials and combinations in humans [9]. A reduction has been consistently observed in babies receiving antibiotics [6] and in mice exposed to ampicillin and gentamicin [8]. Differently, in adult horses, Bifidobacteriaceae were higher after 3 days of oral metronidazole administration [27]. *Bifidobacterium* is another important genus implied in gut protection and immunologic and inflammatory processes in humans [9]. It is considered as a putative beneficial pro-biotic in horses as well [18], and in this regard, it will be later discussed in this manuscript.

Nevertheless, consistent with the present findings, a shift towards Bacteroidetes—and mainly *Bacteroides*—has been observed on the 11th day after treatment with beta lactams in humans, although they were more metabolically active already since the 6th day [51]. Furthermore, little effect or even an increase of broad-spectrum antimicrobials (namely TMS and oxytetracicline) on *Bacteroides* has been shown in adult horses [20].

The genera *Streptococcus* and *Ruminococcus* tended to increase with the combination of antibiotics used in this study. Albeit *Streptococcus* spp. also figures among the most sensitive to several classes of antibiotics used in humans, and *Ruminococcus* decreased in mice treated with ampicillin and gentamicin [8], it was slightly or not at all affected from broad-spectrum antimicrobials (namely TMS and oxytetracycline) in adult horses [20].

Overall, antimicrobial administration in neonatal foals tends to decrease cellulolytic and fibrolytic bacteria, and the microbiota disruption might lead to a proliferation of pathogenic bacteria, which is in line to what it has been described in adult horses [19].

### 4.2. Fecal Microbiota in Neonatal Foals at Hospital Discharge

With regard to the findings at discharge, despite only a few days passing since the end of the treatment, as the foals were rebuilding up their gut flora, a trend to gain a higher microbial diversity was already observed.

The literature is relatively consistent across species in reporting that the gut microbiota has a degree of resilience, yet a variable amount of time is needed to recover the pre-treatment or health-associated gut ecosystem. In particular, in babies, an incomplete recovery of the gut microbiota has been observed months after cessation of the treatment, and after weeks in mice [6]. In adult horses, overall, it seems that the gut microbiota needs up to approximatively 30 days to recover a state similar to the baseline, albeit with still detectable differences, after antimicrobials administration [18,19,20,26].

In the present study, a new increase in microbial diversity seems to occur faster than in adult horses, as the earliest samples were only two days from the end of the treatment. Costa et al. [25] suggested that in neonate foals, the microbiota is already highly dynamic, thus, its rapid physiologic changing might lead to a more rapid recovery even after external perturbations. The recovery period (Te–Td) was not homogeneous. Although it is unlikely that the shorter period included was enough for fecal microbiota recovery (2 days for foal GRA), no comparison or clusterization between foals was possible to confirm it. On a speculative ground, looking at relative genera abundances across individuals, the foal GRA appeared to show more evident differences at Td with respect to the others (Figure 1).

In Figure 4, it is depicted how some of the microorganisms (i.e., *Eubacterium*) which were characteristic of the pre-treatment samples have reappeared at discharge. This is consistent with a trend toward reestablishing an eubiotic setting of the gut ecosystem after recovery from antimicrobials, where fibrolytic and SCFA productor taxa are dominant over pathobionts. In this contest, *Akkermansia* figures also as a leading microorganism at discharge.

Noteworthy, since microbiota in neonates is not yet fully developed and established, it is reasonable that the effects of antibiotics treatment cannot be expressed as a measure of return to the pre-treatment state. In the PCoA analysis, a shift of the ellipse is visible, meaning that bacterial communities are different between the pre-treatment time and the time of discharge from the hospital in our population. Furthermore, a higher convergence of the samples is depicted at discharge.

This may be a selective effect of the antimicrobials administration itself. Indeed, the perturbations induced may cause a permanent shift in the ecosystem or lead toward a different developmental trajectory. In humans, the sequence of bacterial colonization is important in shaping the gut microbiome, so it has been suggested that early antimicrobial-associated changes may have long term effects [7]. Nevertheless, we might also speculate on the role of the shared environment among foals after the hospitalization period with respect to their diverse provenience at the time of enrollment.

### 4.3. Limitations and Potential Confounding Factors

Overall, the small number of enrolled foals represents a main limitation of the study and might have impaired the ability to detect significant changes in the fecal microbiota associated to antimicrobial administration. Regardless, as it was already suggested by Costa et al. [19], the clinical relevance of changes might not always correspond to their magnitude (i.e., less evident changes might be more clinically relevant), and it needs to be assessed in specific populations and settings. The inability to standardize factors such as antimicrobials administration duration or recovery period, or to group foals based on homogeneous intervals, also represent a weakness of the study.

Furthermore, many confounding factors may have played on the results observed in the present population, rendering an interpretation of the findings with regard to genuine effects of antimicrobials is not straightforward. First of all, as mentioned above, a leading effect of age-related change in the gut microbiota. Although we attempted to reduce some of the age-related variability by enrolling only foals within 48 h of life, the neonatal gut microbiota is characterized by a highly variable and dynamic flora, following its trajectory toward an adult-like gut microbiota, with rapid changes happening in the period encompassed by the study [21,22,23,24,25].

If we compare the pre-treatment gut microbiota composition with a previous study conducted on healthy neonatal foals born at the same hospital, although there is concordance on the main phyla represented, they differ in their relative abundance, in particular, for Fimicutes and Proteobacteria [23], which may be attributed, at least partially, to the pathological conditions of the foals in this study. At the taxonomic level of family, divergences are even more evident. Indeed, the mean relative abundances of Enterococcaceae and Enterobacteriaceae are much superior in the present study with respect to the findings by Quercia et al. (2019) (13% vs. 2% and 18% vs. 9%, respectively) [23]. However, all the baseline samples were from meconium in the study by Quercia et al. (2019), while in the present one, there were some of meconium and some feces at Ta, and they may reflect the shift from communities that are characteristic of meconium toward milk-associated taxa [23]. From the 3rd to 5th day, with the normal expression of coprophagy by neonatal foals, the intestinal microbiota composition starts to converge toward the adults’ core microbiota, with the acquisition of fiber-fermenters microorganisms, such as *Blautia* and *Ruminococcus* [23], and this trend is reflected in samples at Td in this study.

As it is reported in adult equines [19], many other animal-related factors may contribute to the high variability of gut microbiota among foals, such as breed, sex and environments. Recently, breed has been shown to exert limited effects on the faecal microbiota in adult horses [57]. While human beings coming from different areas house different microbial populations [58], Liu et al. (2020) did not find a difference between farms in the overall intestinal microbiota of foals slightly older than the present population [29]. Maybe most importantly, they were in different pathological conditions, which prompted a treatment for either prophylactic or therapeutic purposes. Many pathological conditions have been associated with gut microbiota perturbations in adult horses and foals [59,60,61,62,63,64,65]. To the authors’ knowledge, while it is very likely, it is unknown whether and to what extent there is an association of preexisting conditions encountered in this study on the fecal microbiota. Conversely, it is also undetermined from this study whether the same combination of antimicrobials might impact the microbiota differently depending on the conditions (e.g., different pharmacodynamics) [17]. In the present study, the low number of the sample precluded any comparison between foals grouped by condition. These aspects should be investigated in future studies on the ground of antimicrobials selection, especially in the contest of a better antimicrobial stewardship.

In addition to the inherent animal factors, there are a number of other factors related to the clinical condition and hospitalization to consider. All the foals received a probiotic product during the antimicrobial treatment or even the whole hospitalization. The effects of probiotics on the microbiota changes could not be determined nor discriminated in the present population, and further studies are needed to compare foals administered broad-spectrum antimicrobials with or without probiotics supplementation. Overall, the evidence for probiotics use in horses and foals is not consistent [18,19,28,66]. It was previously hypothesized that probiotics might be of more benefit in foals than adults [67], however, Schoster et al. (2016) [68] did not detect an increase in *Lactobacillus* and *Bifidobacterium* in neonatal foals receiving a multistrain probiotics product. In this study, a different product was used, and there was a tendency to increase in *Lactobacillus* and *Bifidobacterium* throughout time, which, as already discussed, is in contrast with findings associated with antimicrobials exposure in most of the human literature [6,8,9].

In spite of the administration of probiotics, four out of the seven foals developed a mild diarrhea either during or after the antimicrobial treatment. Foal diarrhea shows a high frequency in the first 6 months, and often the etiology remains undetermined [69]. As previously observed, it is difficult to establish what comes first, disease or altered microbiome [19]. Regardless of the cause, diarrhea is accompanied by a general reduction in microbial diversity, and a consistent reduction in Lachnospiraceae and Ruminococcaceae in neonatal foals [64]. Both families tended to have a lower mean abundance after the antimicrobial treatment and increased again afterwards in our population.

Although it is speculative, we think it is intriguing that the four foals developing a diarrhea were the same ones which received prophylactically omeprazole. Omeprazole administration has been associated with a higher risk of diarrhea in neonatal foals [70]. Available data in adult horses suggest that proton-pump inhibitors (PPIs) administration might not have significant effects on intestinal microbiota [71,72], but this is still undetermined in foals.

Notably, three of the four foals received one to three fecal microbiota transplantations (FMT) during the course of diarrhea, which was considered effective in all cases. While the use of FMT in adult horses with diarrhea has been recently evaluated by means of NGS [73], to the authors knowledge, there is no peer-reviewed literature on the efficacy of FMT in neonatal foals [74]. McKinney et al. (2021) reported that FMT proved an effective treatment to reduce diarrhea severity in horses with colitis and to improve microbiome diversity [73]. While it is very likely that FMT had influenced the findings and represent a main confounding factor, an effect of FMT on fecal microbiota dynamic was not possible to be discriminated in this study. Furthermore, the donor composition was not determined for comparison. The contribution of FMT to the results in this study remain to be elucidated.

A last factor the authors think is worth commenting on is the milk source, which was not uniform among foals. In the human literature, a less diverse, with less potential pathobionts, has been reported in breast-fed babies with respect to formula-fed babies [75]. However, the type of feeding (breast vs. formula feeding) did not result in significant differences at the phylum level in infants who received or did not received antibiotics, except for formula-fed infants acquiring more *Enterococcus faecalis* over time [7]. To the authors knowledge, there are no data available for foals.

Overall, there are multiple potential confounding factors on the findings, which could not be controlled in the setting of the present study, should be evaluated by comparison of larger sample sizes where clustering of subjects by these factors would be possible. As it is in humans, the large inter-individual variability in the microbiota composition prompts multiomic approaches to explore its functionality and host-interaction [6].

## 5. Conclusions

Overall, the findings of this small-scale study are not conclusive but tend to be in line with the acknowledged antimicrobial-associated gut microbiota modifications in human medicine. A trend to a generalized loss of alpha diversity was observed after IV ampicillin-aminoglycoside administration and supplementation with probiotics. A degree of unbalance of the relative abundances toward pathobionts over beneficial SCFAs producers was observed but could not be confirmed. This general picture tended to revert toward a more eubiotic setting after a few days of recovery. A relatively high beta diversity observed at this time might be expected, due to the highly dynamic changes occurring in the gut microbiota in foals of this age. It is possible that in this population of foals, the effect of antimicrobials is strongly influenced and possibly overshadowed by the changes that occur over time in the developing gut microbiota. While multiple limitations and confounding factors prevent the authors from drawing conclusions, especially in terms of changes to the microbial taxa, these preliminary findings suggest a general trend characterized by a loss of fecal microbial diversity after a short course of broad-spectrum antimicrobials administration followed by a rapid increase after antimicrobials have been interrupted. Of note, the findings are influenced by the use of probiotics, and whether the changes would be consistent in antimicrobial-administered but not supplemented foals remains to be elucidated. The effects of a different treatment duration and recovery period could be different. The relationship between fecal microbiota and animal-inherent factors and pathological conditions, the impact of FMT as well as other external factors related to hospitalization warrant investigations in larger populations and homogenous groups of foals.

## Figures and Tables

**Table 1:** Demographic data, clinical diagnosis and summary of treatments in seven hospitalized neonatal foals treated with broad-spectrum antimicrobials within 48 h after birth. Acronyms of foals’ names are given for identification (CM, GRA, FDT, GR, PIL, RH, TR).

Foal	CM	GRA	FDT	GR	PIL	RH	TR
Breed	Arab	WB	WB	Stb	Stb	Stb	Stb
Place of birth	Farm	Farm	Farm	VTH	VTH	VTH	VTH
Ta (age in hours)	30	42	10	0	18	18	18
IgG (mg/dL)	135	426	1535	827	337	677	135
Diagnosis	Sepsis, Dysmaturiy, FPT	PAS, FPT	PAS	PAS	FPT	FPT	FPT
T/P antimicrobials	T	P	P	P	P	P	P
Antimicrobials association	ampicillin/ gentamicin	ampicillin/ amikacin	ampicillin/ gentamicin	ampicillin/ amikacin	ampicillin/ amikacin	ampicillin/ amikacin	ampicillin/ amikacin
Antimicrobials treatm duration (days)	12	8	5	6	5	6	8
Te–Td (days)	3	2	9	3	3	5	5
Diarrhoea	Yes	Yes	Yes	No	No	No	Yes
FMT n°; timeframe	3; Ta–Te	No	2; Te–Td	No	No	No	1; Ta–Te (F)
Other treatments	ome/sucr	ome/sucr	ome/sucr	sucr	/	/	ome/sucr
Source of milk	M/R	M	M/R	M	M	M	M/R/F

Arab: Arabian; WB: Warmblood; Stb: Standardbred. VTH: Veterinary Teaching Hospital. Ta: time (hours) of antimicrobial treatment start; Te: next day after the end of antimicrobial treatment; Td: day of hospital discharge. FPT: failure of passive transfer of immunity; PAS: Perinatal Asphyxia Syndrome; T: therapeutic antimicrobial treatment; P: prophylactic antimicrobial treatment; FMT: fecal microbial transplantation, number of treatments (n°) and timeframe of treatment/s during hospitalization; ome: omeprazole; sucr: sucralfate; M: mare; R: milk replacer; F: foster mare.

## Data Availability

Amplicon sequences were deposited in the MG-Rast database (http://metagenomics.anl.gov/mgmain.html?mgpage=project&prject=59129e34be6d67703833303632, accessed on 20 May 2021).

## Supplementary Materials

The following are available online at https://www.mdpi.com/article/10.3390/ani11082283/s1, Table S1: Mean relative abundance of taxa correspondence for OTUs.

## References

[1] Theelen M.J., Wilson W.D., Byrne B.A., Edman J.M., Kass P.H., Magdesian K.G. (2019). Initial antimicrobial treatment of foals with sepsis: Do our choices make a difference?. Vet. J..

[2] Floyd E.F., Easton-Jones C.A., Theelen M.J.P. (2021). Systemic antimicrobial therapy in foals. Equine Vet. Educ..

[3] Magdesian K.G. (2017). Antimicrobial pharmacology for the neonatal foal. Vet. Clin. Equine Pract..

[4] Magdesian K.G. (2005). Neonatal Foal Diarrhea. Vet. Clin. Equine Pract..

[5] Rodríguez J.M., Murphy K., Stanton C., Ross R.P., Kober O.I., Juge N., Avershina E., Collado M.C. (2015). The composition of the gut microbiota throughout life, with an emphasis on early life. Microb. Ecol. Health Dis..

[6] Nogacka A.M., Salazar N., Arboleya S., Suárez M., Fernández N., Solís G., de los Reyes-Gavilán C.G., Gueimonde M. (2018). Early microbiota, antibiotics and health. Cell. Mol. Life Sci..

[7] Eck A., Rutten N.B., Singendonk M.M., Rijkers G.T., Savelkoul P.H., Meijssen C.B., Crijns C.E., Oudshoorn J.H., Budding A.E., Vlieger A.M. (2020). Neonatal microbiota development and the effect of early life antibiotics are determined by two distinct settler types. PLoS ONE.

[8] Rosa C.P., Brancaglion G.A., Miyauchi-Tavares T.M., Corsetti P.P., de Almeida L.A. (2018). Antibiotic-induced dysbiosis effects on the murine gastrointestinal tract and their systemic re-percussions. Life Sci..

[9] Ferrer M., Méndez-García C., Rojo D., Barbas C., Moya A. (2017). Antibiotic use and microbiome function. Biochem. Pharmacol..

[10] Ling Z., Liu X., Jia X., Cheng Y., Luo Y., Yuan L., Wang Y., Xiang C. (2014). Impacts of infection with different toxigenic Clostridium difficile strains on faecal microbiota in children. Sci. Rep..

[11] Tanaka S., Kobayashi T., Songjinda P., Tateyama A., Tsubouchi M., Kiyohara C., Shi-rakawa T., Sonomoto K., Nakayama J. (2009). Influence of antibiotic exposure in the early post-natal period on the development of intestinal microbiota. FEMS Immunol. Med. Microbiol..

[12] De La Cochetière M.F., Durand T., Lalande V., Petit J.C., Potel G., Beaugerie L. (2008). Effect of antibiotic therapy on human fecal microbiota and the relation to the development of Clostridium difficile. Microb. Ecol..

[13] Barbosa T.M., Levy S.B. (2000). The impact of antibiotic use on resistance development and persistence. Drug Resist. Updat..

[14] Robinson C.J., Young V.B. (2010). Antibiotic administration alters the community structure of the gastrointestinal microbiota. Gut Microbes.

[15] Ianiro G., Tilg H., Gasbarrini A. (2016). Antibiotics as deep modulators of gut microbiota: Between good and evil. Gut.

[16] Iizumi T., Battaglia T., Ruiz V., Perez G.I.P. (2017). Gut microbiome and antibiotics. Arch. Med. Res..

[17] Kauter A., Epping L., Semmler T., Antao E.M., Kannapin D., Stoeckle S.D., Gehlen H., Lübke-Becker A., Günther S., Wiele L.H. (2019). The gut microbiome of horses: Current research on equine enteral microbiota and future perspectives. Anim. Microbiome.

[18] Garber A., Hastie P., Murray J.A. (2020). Factors influencing equine gut microbiota: Current knowledge. J. Equine Vet. Sci..

[19] Costa M.C., Stämpfli H.R., Arroyo L.G., Allen-Vercoe E., Gomes R.G., Weese J.S. (2015). Changes in the equine fecal microbiota associated with the use of systemic antimicrobial drugs. BMC Vet. Res..

[20] De La Torre U., Henderson J.D., Furtado K.L., Pedroja M., Elenamarie O.M., Mora A., Pechanec M.Y., Maga E.A., Mienaltowski M.J. (2019). Utilizing the fecal microbiota to understand foal gut transitions from birth to weaning. PLoS ONE.

[21] Lindenberg F., Krych L., Kot W., Fielden J., Frøkiær H., van Galen G., Nielsen D.S., Hansen A.K. (2019). Development of the equine gut microbiota. Sci. Rep..

[22] Quercia S., Freccero F., Castagnetti C., Soverini M., Turroni S., Biagi E., Rampelli S., Lanci A., Mariella J., Chinellato E. (2019). Early colonisation and tem-poral dynamics of the gut microbial ecosystem in Standardbred foals. Equine Vet. J..

[23] Husso A., Jalanka J., Alipour M.J., Huhti P., Kareskoski M., Pessa-Morikawa T., Iivan-ainen A., Niku M. (2020). The composition of the perinatal intestinal microbiota in horse. Sci. Rep..

[24] Costa M.C., Stämpfli H.R., Allen-Vercoe E., Weese J.S. (2016). Development of the faecal micro-biota in foals. Equine Vet. J..

[25] Collinet A., Grimm P., Julliand S., Julliand V. (2019). Oral administration of antibiotics alters fecal ecosystem of adult horses in the long-term. J. Equine Vet. Sci..

[26] Arnold C.E., Isaiah A., Pilla R., Lidbury J., Coverdale J.S., Callaway T.R., Lawhon S.D., Steiner J., Suchodolski J.S. (2020). The cecal and fecal microbiomes and metabolomes of horses before and after metronidazole administration. PLoS ONE.

[27] Collinet A., Grimm P., Julliand S., Julliand V. (2021). Multidimensional approach for investigating the effects of an antibiotic–probiotic combination on the equine hindgut ecosystem and microbial fibrolysis. Front. Microbiol..

[28] Knych H.K., Magdesian K.G. (2021). Equine antimicrobial therapy: Current and past issues facing practitioners. J. Vet. Pharmacol. Ther..

[29] Liu Y., Bailey K.E., Dyall-Smith M., Marenda M.S., Hardefeldt L.Y., Browning G.F., Gilkerson J.R., Billman-Jacobe H. (2021). Faecal microbiota and antimicrobial resistance gene profiles of healthy foals. Equine Vet. J..

[30] Álvarez–Narváez S., Berghaus L.J., Morris E.R.A., Willingham-Lane J.M., Slovis N.M., Giguere S., Cohen N.D. (2020). A common practice of widespread antimicrobial use in horse pro-duction promotes multi-drug resistance. Sci. Rep..

[31] Vaala W.E., House J.K., Madigan J.E., Smith B.P. (2002). Initial management and physical examination of the neonate. Large Animal Internal Medicine.

[32] Giguére S., Polkes A.C. (2005). Immunologic disorders of neonatal foals. Vet. Clin. Equine Pract..

[33] Castagnetti C., Pirrone A., Mariella J., Mari G. (2010). Venous blood lactate evaluation in equine neonatal intensive care. Theriogenology.

[34] Wong D.M., Wilkins P.A. (2015). Defining the Systemic Inflammatory Response Syndrome in Equine Neonates. Vet. Clin. Equine Pract..

[35] Toribio R.E. (2019). Equine Neonatal Encephalopathy: Facts, Evidence, and Opinions. Vet. Clin. Equine Pract..

[36] Knottenbelt D.C., Holdstock N., Madigan J.E. (2004). Equine Neonatology Medicine and Surgery.

[37] Biagi E., Franceschi C., Rampelli S., Severgnini M., Ostan R., Turroni S., Consolandi C., Quercia S., Scurti M., Monti D. (2016). Gut Microbiota and Extreme Longevity. Curr. Biol..

[38] Yu Z., Morrison M. (2004). Improved extraction of PCR-quality community DNA from digesta and fecal samples. Biotechniques.

[39] Klindworth A., Pruesse E., Schweer T., Peplies J., Quast C., Horn M., Glöckner F.O. (2013). Evaluation of general 16S ribosomal RNA gene PCR primers for classical and next-generation sequencing-based diversity studies. Nucleic Acids Res..

[40] Candela M., Biagi E., Soverini M., Consolandi C., Quercia S., Severgnini M., Peano C., Turroni S., Rampelli S., Pozzilli P. (2016). Modulation of gut microbiota dysbioses in type 2 diabetic patients by macrobiotic Ma-Pi 2 diet. Br. J. Nutr..

[41] Biagi E., Quercia S., Aceti A., Berghetti I., Rampelli S., Turroni S., Faldella G., Candela M., Brigidi P., Corvaglia L. (2017). Bacterial sharing between the ecosystems of mother’s milk and infant’s mouth and gut. Front. Miocrobiol..

[42] Caporaso J.G., Kuczynski J., Stombaugh J., Bittinger K., Bushman F.D., Costello E.K., Fierer N., Peña A.G., Goodrich J.K., Gordon J.I. (2010). QIIME allows analysis of high-throughput community sequencing data. Nat. Methods.

[43] Edgar R.C. (2010). Search and clustering orders of magnitude faster than BLAST. Bioinformatics.

[44] Haas B.J., Gevers D., Earl A.M., Feldgarden M., Ward D.V., Giannoukos G., Ciulla D., Tabbaa D., Highlander S.K., Sodergren E. (2011). Chimeric 16S rRNA sequence formation and detection in Sanger and 454-pyrosequenced PCR amplicons. Genome Res..

[45] McMurdie P.J., Holmes S. (2013). Phyloseq: An R package for reproducible interactive analysis and graphics of microbiome census data. PLoS ONE.

[46] Callahan B.J., Sankaran K., Fukuyama J.A., McMurdie P.J., Holmes S.P. (2016). Bioconductor workflow for microbiome data analysis: From raw reads to community analyses. F1000Research.

[47] Anderson M.J., Walsh D.C.I. (2013). PERMANOVA, ANOSIM, and the Mantel test in the face of heterogeneous dispersions: What null hypothesis are you testing?. Ecol. Monogr..

[48] Oksanen J., Blanchet F.G., Kindt R., Legendre P., Minchin P.R., O’hara R.B., Oksanen M.J. (2013). Package ‘vegan’. Community Ecology Package, Version. http://sortie-admin.readyhosting.com/lme/R%20Packages/vegan.pdf.

[49] Wickham H. (2011). Ggplot2: Elegant graphics for data analysis. J. R. Stat. Soc. Ser. A.

[50] Love M.I., Huber W., Anders S. (2014). Moderated estimation of fold change and dispersion for RNA-seq data with DESeq2. Genome Biol..

[51] Pérez-Cobas A.E., Gosalbes M.J., Friedrichs A., Knecht H., Artacho A., Eismann K., Otto W., Rojo D., Bargiela R., von Bergen M. (2014). Gut microbiota disturbance during antibiotic therapy: A multiomic approach. Gut.

[52] Greenwood C., Morrow A.L., Lagomarcino A.J., Altaye M., Taft D.H., Yu Z., Newburg D.S., Ward D.V., Schibler K.R. (2014). Early empiric antibiotic use in preterm infants is associated with lower bacterial diversity and higher relative abundance of Enterobacter. J. Pediatr..

[53] Collado M.C., Derrien M., Isolauri E., de Vos W.M., Salminen S. (2007). Intestinal integrity and *Akkermansia muciniphila*, a mucin-degrading member of the intestinal microbiota present in infants, adults, and the elderly. Appl. Environ. Microbiol..

[54] Derrien M., Collado M.C., Ben-Amor K., Salminen S., de Vos W.M. (2008). The Mucin degrader *Akkermansia muciniphila* is an abundant resident of the human intestinal tract. Appl. Environ. Microbiol..

[55] Ottman N., Geerlings S.Y., Aalvink S., de Vos W.M., Belzer C. (2017). Action and function of *Akkermansia muciniphila* in microbiome ecology, health and disease. Best Pract. Res. Clin. Gastroenterol..

[56] Arboleya S., Sánchez B., Solís G., Fernández N., Suárez M., Hernández-Barranco A.M., Milani C., Margolles A., de los Reyes-Gavilán C.G., Ventura M. (2016). Impact of prematurity and perinatal antibiotics on the developing intestinal microbiota: A functional inference study. Int. J. Mol. Sci..

[57] Massacci F.R., Clark A., Ruet A., Lansade L., Costa M., Mach N. (2020). Inter-breed diversity and temporal dynamics of the faecal microbiota in healthy horses. J. Anim. Breed. Genet..

[58] Chong C., Bloomfield F., O’Sullivan J. (2018). Factors Affecting Gastrointestinal Microbiome Development in Neonates. Nutrients.

[59] Garrett L.A., Brown R., Poxton I.R. (2002). A comparative study of the intestinal microbiota of healthy horses and those suffering from equine grass sickness. Vet. Microbiol..

[60] Costa M.C., Arroyo L.G., Allen-Vercoe E., Stampfli H.R., Kim P.T., Sturgeon A., Weese S.J. (2012). Comparison of the fecal microbiota of healthy horses and horses with colitis by high throughput sequencing of the V3-V5 region of the 16S rRNA gene. PLoS ONE.

[61] Moreau M.M., Eades S.C., Reinemeyer C.R., Fugaro M.N., Onishi J.C. (2014). Illumina sequencing of the V4 hypervariable region 16S rRNA gene reveals extensive changes in bacterial communities in the cecum following carbohydrate oral infusion and development of early-stage acute laminitis in the horse. Vet. Microbiol..

[62] Weese J.S., Holcombe S.J., Embertson R.M., Kurtz K.A., Roessner H.A., Jalali M., Wismer S.E. (2015). Changes in the faecal microbiota of mares precede the development of post partum colic. Equine Vet. J..

[63] Elzinga S.E., Weese J.S., Adams A.A. (2016). Comparison of the fecal microbiota in horses with equine metabolic syndrome and metabolically normal controls fed a similar all-forage diet. J. Equine Vet. Sci..

[64] Schoster A., Staempfli H.R., Guardabassi L.G., Jalali M., Weese J.S. (2017). Comparison of the fecal bacterial microbiota of healthy and diarrheic foals at two and four weeks of life. BMC Vet. Res..

[65] Stewart H.L., Southwood L.L., Indugu N., Vecchiarelli B., Engiles J.B., Pitta D. (2019). Differences in the equine faecal microbiota between horses presenting to a tertiary referral hospital for colic compared with an elective surgical procedure. Equine Vet. J..

[66] Schoster A. (2018). Probiotic Use in Equine Gastrointestinal Disease. Vet. Clin. Equine Pract..

[67] Weese J.S., Maureen E.C., Anderson A.L., Monteith G.J. (2003). Preliminary investigation of the probiotic potential of Lactobacillus rhamnosus strain GG in horses: Fecal recovery following oral administration and safety. Can. Vet. J..

[68] Schoster A., Guardabassi L., Staempfli H.R., Abrahams M., Jalali M., Weese J.S. (2016). The Longitudinal Effect of a Multi-Strain Probiotic on the Intestinal Bacterial Microbiota of Neonatal Foals. Equine Vet. J..

[69] Schoster A., Staempfli H.R., Abrahams M., Jalali M., Weese J.S., Guardabassi L. (2015). Effect of a probiotic on prevention of diarrhea and Clostridium difficile and Clostridium perfringens shedding in foals. JVIM.

[70] Furr M., Cohen N.D., Axon J.E., Sanchez L.C., Pantaleon L., Haggett E., Campbell R., Tennent-Brown B. (2012). Treatment with histamine-type 2 receptor antagonists and omeprazole increase the risk of diarrhoea in neonatal foals treated in intensive care units. Equine Vet. J..

[71] Cerri S., Taminiau B., de Lusancay A.H.C., Lecoq L., Amory H., Daube G., Cesarini C. (2020). Effect of oral administration of omeprazole on the microbiota of the gastric glandular mucosa and feces of healthy horses. JVIM.

[72] Tyma J.F., Epstein K.L., Whitfield-Cargile C.M., Cohen N.D., Giguère S. (2019). Investigation of effects of omeprazole on the fecal and gastric microbiota of healthy adult horses. Am. J. Vet. Res..

[73] McKinney C.A., Bedenice D., Pacheco A.P., Oliveira B.C., Paradis M.R., Mazan M., Widmer G. (2021). Assessment of clinical and microbiota responses to fecal microbial transplantation in adult horses with diarrhea. PLoS ONE.

[74] Mullen K.R., Yasuda K., Divers T.J., Weese J.S. (2018). Equine faecal microbiota transplant: Current knowledge, proposed guidelines and future directions. Equine Vet. Educ..

[75] Milani C., Duranti S., Bottacini F., Casey E., Turroni F., Mahony J., Belzer C., Palacio S.D., Montes S.A., Mancabelli L. (2017). The First Microbial Colonizers of the Human Gut: Composition, Activities, and Health Implications of the Infant Gut Microbiota. MMBR.

