# Supplementary material for: Changes in the Fecal Microbiota Associated with a Broad-Spectrum Antimicrobial Administration in Hospitalized Neonatal Foals with Probiotics Supplementation
Source: Animals (Basel). 2021 Aug 2;11(8):2283. doi: 10.3390/ani11082283 (PMC8388449; doi:10.3390/ani11082283)
Supplement: Supplementary file 1 [file animals-11-02283-s001.zip › animals-1262152-supplementary.pdf]

Table S1. Mean relative abundance of taxa correspondence for OTUs identified in fecal samples in 7 hospitalized neonatal foals before and after broad-spectrum antimicrobial treatment (ampicillin + gentamicin/amikacin. IV).

#OTU	Ta	Te	Td
**Phyla %**			
Actinobacteria	4.48	3.03	6.35
Bacteroidetes	7.55	11.37	7.66
Firmicutes	48.19	38.93	61.58
Fusobacteria	0.14	3.44	0.82
Proteobacteria	31.56	38.31	11.44
TM7	0.12	0.08	0.68
Verrucomicrobia	1.79	3.95	10.28
Other	0.28	0.03	0.37
Unassigned; Other	5.89	0.86	0.82
**Familia %**			
Aerococcaceae	1.88	0.01	0.05
Bacteroidaceae	4.78	11.21	4.72
Bifidobacteriaceae	0.46	1.83	1.99
Carnobacteriaceae	0.12	0.34	0.95
Chitinophagaceae	0.51	0.01	0.00
Clostridiaceae	6.29	2.45	8.32
Members of Clostridiales	0.90	1.17	3.42
Comamonadaceae	0.51	0.00	0.00
Coriobacteriaceae	0.53	0.88	2.94
Corynebacteriaceae	0.46	0.01	0.05
Dermabacteraceae	0.35	0.00	0.00
Enterobacteriaceae	17.55	37.64	10.85
Enterococcaceae	13.23	13.75	7.98
Eubacteriaceae	0.03	0.04	0.37
Erysipelotrichaceae	1.60	1.26	2.20
F16	0.09	0.08	0.68
Fusobacteriaceae	0.14	3.44	0.82
Lachnospiraceae	7.07	4.94	13.00

Lactobacillaceae	1.63	2.56	7.42
Microbacteriaceae	0.68	0.02	0.07
Micrococcaceae	0.31	0.04	1.14
[Mogibacteriaceae]	0.56	0.59	2.69
Moraxellaceae	0.99	0.01	0.04
[Odoribacteraceae]	0.01	0.01	0.41
Peptococcaceae	0.01	0.00	0.01
Peptostreptococcaceae	3.56	1.14	2.47
Pseudomonadaceae	6.40	0.19	0.00
Porphyromonadaceae	0.16	0.09	1.68
Rhizobiaceae	0.67	0.01	0.02
Rikenellaceae	0.09	0.03	0.55
Ruminococcaceae	3.80	3.03	6.47
Sphingobacteriaceae	1.05	0.00	0.00
Sphingomonadaceae	1.16	0.00	0.00
Streptococcaceae	5.32	7.46	4.64
Streptomycetaceae	0.77	0.00	0.01
Turicibacteraceae	0.46	0.00	0.00
Verrucomicrobiaceae	1.76	3.94	10.22
Xanthomonadaceae	2.14	0.01	0.01
Other	6.11	0.94	2.97
Unassigned; Other	5.89	0.86	0.82
**Genera %**			
Acinetobacter	0.95	0.00	0.02
Aerococcus	1.54	0.01	0.01
Akkermansia	1.76	3.94	10.22
Arthrobacter	0.22	0.02	0.96
Bacteroides	4.76	11.21	4.67
Bifidobacterium	0.46	1.83	1.99
Blautia	1.11	0.31	1.35
Brachybacterium	0.35	0.00	0.00
Butyricimonas	0.01	0.01	0.34
Clostridium	1.34	0.20	3.90

Members of Clostridiaceae	4.90	2.22	2.42
Members of Coriobacteriaceae	0.13	0.07	0.47
Coprococcus	0.28	0.06	0.79
Corynebacterium	0.46	0.01	0.05
Curtobacterium	0.50	0.01	0.02
Delftia	0.34	0.00	0.00
Dorea	0.67	0.41	3.52
Eggerthella	0.16	0.79	2.26
Members of Enterobacteriaceae	17.08	35.94	9.73
Enterococcus	13.23	13.75	7.98
Epulopiscium	2.40	0.01	0.07
Erwinia	0.39	0.00	0.00
Members of Erysipelotrichaceae	0.85	0.51	1.42
Eubacterium	0.66	0.69	0.51
Members of F16	0.09	0.08	0.68
Faecalibacterium	0.77	0.01	0.01
Fusobacterium	0.14	3.44	0.82
Klebsiella	0.07	1.35	1.06
Members of Lachnospiraceae	0.81	0.80	3.04
Lactobacillus	1.62	2.54	7.41
Members of Mogibacteriaceae	0.55	0.59	2.64
Oscillospira	0.49	1.01	1.52
Parabacteroides	0.16	0.09	1.67
Members of Peptostreptococcaceae	3.56	1.14	2.45
Pseudomonas	6.33	0.18	0.00
Members of Rikenellaceae	0.09	0.03	0.55
[Ruminococcus]	1.41	3.27	4.16
Ruminococcus	0.81	0.24	1.47
Members of Ruminococcaceae	1.71	1.75	3.38
Sarcina	0.05	0.03	1.94
Sphingobacterium	0.61	0.00	0.00
Sphingomonas	0.98	0.00	0.00
Sediminibacterium	0.51	0.01	0.00

Stenotrophomonas	1.43	0.01	0.01
Streptococcus	5.30	7.44	4.61
Streptomyces	0.77	0.00	0.01
Turicibacter	0.46	0.00	0.00
Members of Xanthomonadaceae	0.63	0.00	0.00
Other	9.31	1.89	5.24
Unassigned; Other	5.89	0.86	0.82

Ta: time (hours) of antimicrobial treatment start; Te: end of antimicrobial treatment; Td: day of hospital discharge.
